# The Pertinent Literature of Enhanced Recovery after Surgery Programs: A Bibliometric Approach

**DOI:** 10.3390/medicina57020172

**Published:** 2021-02-17

**Authors:** Cheng Li, Yang Cheng, Zhao Li, Donara Margaryan, Carsten Perka, Andrej Trampuz

**Affiliations:** 1 Center for Musculoskeletal Surgery (CMSC), Charité-Universitätsmedizin Berlin, Corporate Member of Freie Universität Berlin, Humboldt-Universität zu Berlin and Berlin Institute of Health, 10117 Berlin, Germany; cheng.li@charite.de (C.L.); zhao.li@charite.de (Z.L.); donara.margaryan@charite.de (D.M.); carsten.perka@charite.de (C.P.); 2 Department of Respiratory and Critical Care Medicine, The Fourth Medical College of Peking University, Beijing 100035, China; chengyang@jst-hosp.com.cn

**Keywords:** enhanced recovery after surgery, complications, disease management, patient outcome assessment, bibliometric analysis

## Abstract

*Background and Objectives:* The programs of enhanced recovery after surgery are the new revolution in surgical departments; however, features of this concept have not been systematically explored. Therefore, the purpose of this study was to explore Enhanced recovery after surgery (ERAS)-related research using bibliometric analysis. *Materials and Methods:* The search strategy of ERAS programs was conducted in the Web of Science database. Bibliometric analysis was further performed by Excel and Bibliometrix software. The relationship between citation counts and Mendeley readers was assessed by linear regression analysis. *Results:* 8539 studies from 1994–2019 were included in the present research, with reporting studies originating from 91 countries using 18 languages. The United States (US) published the greatest number of articles. International cooperation was discovered in 82 countries, with the most cooperative country being the United Kingdom. Henrik Kehlet was found to have published the highest number of studies. The journal Anesthesia and Analgesia had the largest number of articles. Linear regression analysis presented a strong positive correlation between citations and Mendeley readers. Most research was related to gastrointestinal surgery in this field. *Conclusion:* This bibliometric analysis shows the current status of ERAS programs from multiple perspectives, and it provides reference and guidance to scholars for further research.

## 1. Introduction

Enhanced recovery after surgery (ERAS) is a series of effective perioperative interventions under the instruction of a multidisciplinary team, thus improving the surgical patients’ experiences both psychologically and physiologically [1,2]. Compared with the traditional perspective, the ERAS pathway is the new revolution for surgery, as it significantly decreases surgical complications, hospital stay, overall medical charges, and patient discomfort [3,4,5]. ERAS has a broad prospect of perioperative application, and an increasing number of surgery-related guidelines have been published in recent years. Meanwhile, ERAS management is complex and involves multiple measures, with controversy remaining in its clinical application. Therefore, ERAS programs still need to be continually refined and optimized in practice [6,7]. To improve and guide further research, it is necessary to investigate the current developmental status and research hotspots of ERAS programs in different surgical specialties.

Scientific publication is the core of discipline development and scholarly communication, and the creation of clinical practice guidelines also depends on the strong evidence from the literature [8,9,10]. Bibliometric analysis is a method for discovering the characteristics of a research subject to understand the current status and trend through the literature, and it is commonly used in the medical field [11,12,13,14,15]. Unfortunately, as one of the hot topics in the surgical field, ERAS has not been assessed.

Citations are a significant indicator for the assessment of literature quality in bibliometric studies [16]. However, with the development of internet technology, alternative indicators have also become a promising literature measurement. Mendeley is one of the most frequently used reference management softwares for scholars that is available locally or online. Mendeley readers were recorded by the number of users who saved the publication in their Mendeley library. This is a commonly used indicator to analyze the relationship with citations in bibliometric studies [17,18,19]. Previous studies found a strong positive correlation between Mendeley readers and citations [20,21]. The investigation of the relationship between citation counts and alternative indicators could make up for the deficiency from fewer citations in the early stage and provide another means of helping scholars to assess the value of the literature. However, no literature has assessed the relationship between Mendeley readers and citations in ERAS studies.

The aim of the present study was: (1) to find the study characteristics in ERAS programs from the country, author, journal, type of surgery, and research topic applied by bibliometric analysis; (2) to identify the relationship between citation counts and Mendeley readers in ERAS-related research.

## 2. Materials and Methods

### 2.1. Data Sources and Search Strategy

The online database of Web of Science was systematically searched using the following search terms: (Enhanced recovery after surgery OR Enhanced recovery program OR Enhanced recovery pathways OR Accelerated rehabilitation OR Fast track surgery).

### 2.2. Data Acquisition

Total records from the Web of Science were exported to plain text and Excel format. Excel (Microsoft, Redmond, WA, USA) and Bibliometrix (University of Naples Federico II, Naples, Italy) softwares were used to analyze the results, which included author, document type, number of citations, country, journal quality, digital object identifier, journal impact factor, publication source, institution, abstract, keywords, article title, language, type of surgery, and publication year [22]. The data of Mendeley reader counts were identified from the official website of Mendeley using the article title or digital object identifier.

### 2.3. Statistical Analysis

Linear regression analysis was performed using the R language (R Core Team, Newark, NJ, USA) to examine the relationship between citations and Mendeley readers and determine the correlation coefficient.

## 3. Results

### 3.1. Publication Output

**Figure 1:**
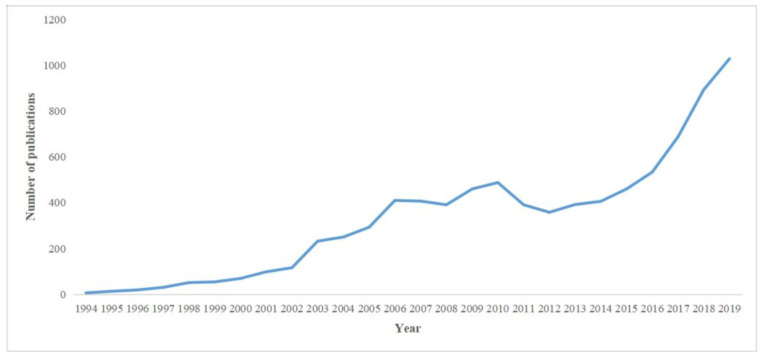
Annual output between 1994 and 2019.

A total of 8539 papers were associated with ERAS programs between 1994 and 2019. Figure 1 shows the annual number of publications and demonstrates an upward trend in recent years, with a large percentage of publications presented during 2014–2019 (4011; 46.9%). The overall publications included 7263 (85.1%) original articles, 968 (11.3%) systematic reviews, and 308 (3.6%) proceedings papers. All included studies were published in 18 languages, with most in English (8156; 95.5%), followed by 233 (2.7%) in German, and 71 (0.8%) in French (Table 1).

### 3.2. The Status of Global Contributions and Collaborations

**Figure 2:**
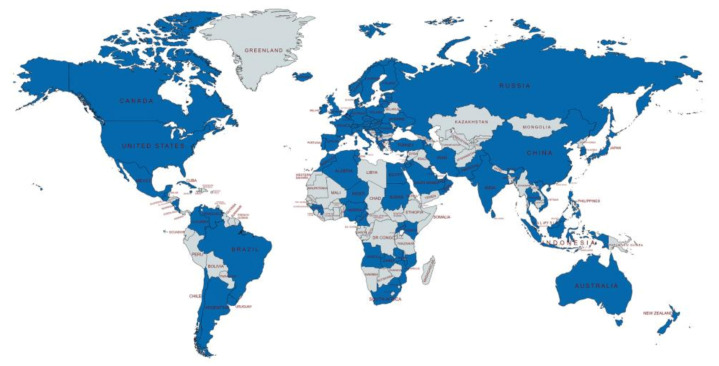
Global distribution of Enhanced recovery after surgery (ERAS)-relevant studies.

Globally, 91 countries participated in the relevant research of the ERAS program (Figure 2). The United States (US) contributed the most in this field with 2717 (31.8%) papers and also had the highest total citation count (107,682). The United Kingdom (UK) ranked second (818; 9.6%) and China third (663, 7.8%) in the number of publications (Table 2).

Eighty-two countries participated in the collaboration. The UK had the largest number of cooperations with other countries (52), followed by the US (50) and The Netherlands (44). The most frequent collaborations were between the US and Canada (132), followed by the US and the UK (98), and the US and Germany (71). Table 3 shows the number of collaborations between countries, which occurred on more than 30 occasions. Approximately 71.9% (22) of collaborations were from the top 10 most contributing countries.

### 3.3. Author Contributions

**Figure 3:**
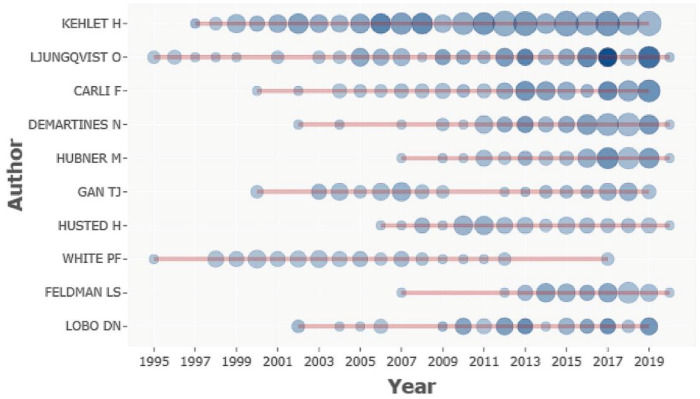
Top 10 authors’ productions over time.

A total of 33,762 authors contributed to ERAS-related research. The percentage of single-authored documents was 3.1% (265). In the top 10 most contributing author list, Henrik Kehlet, from the Department of Surgical Pathophysiology, had the highest number of publications (261), citations (21,497), and h-index (74). The second and third most productive authors in this field are Olle Ljungqvist and Francesco Carli (99 and 88, respectively). The top 10 most productive authors come from seven different institutions, with more than half of the authors being from European countries (Table 4). Figure 3 presented the top 10 authors’ average outputs between 1994 and 2019.

### 3.4. Journal Information and Type of Surgery

**Figure 4:**
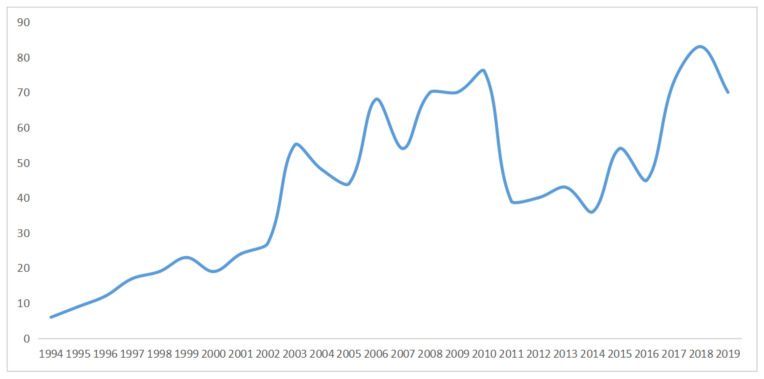
Year of first publication from the journals.

ERAS-related research was published in 1121 journals, and 1014 journals had an impact factor in 2019. Around 27.7% of journals (281) were ranked in the first quartile, 25.4% (258) in the second quartile, 23.9% (243) in the third quartile, and 22.8% (232) in the fourth quartile. Anesthesia and Analgesia had the greatest number of papers (358), followed by the British Journal of Anaesthesia (189), and Acta Anaesthesiologica Scandinavica (147). The rank list of the top 10 journals shows The New England Journal of Medicine has the highest impact factor (74.699), followed by Lancet (60.392), and The Journal of the American Medical Association (45.54; Table 5). Figure 4 presents the year of the journal’s first publication in ERAS, with most journals appearing in 2018 (83), followed by 2010 (76) and 2017 (73).

The surgical information was identified from 6920 papers with 11 clinical departments. Gastrointestinal surgery had the greatest number of papers with ERAS programs (2162), followed by orthopedic surgery (1740), and cardiac surgery (608). In these 11 clinical departments, most journals were from the first quartile (8; Table 6).

### 3.5. Citations and Mendeley Readers

Of the articles, 8104 had at least one citation. Table 7 shows the top five most cited papers in ERAS, with a range of citations between 970 and 1923 and all publications ranked in the first quartile of the medical journal. In the Mendeley database, Mendeley readers of 8079 publications could be found. Linear regression analysis revealed a significant positive correlation (Pearson r: r = 0.7008; *p* < 0.001) between citations and Mendeley readers.

### 3.6. Research Topic

The research topic was identified by thematic maps in Bibliometrix. Ninety-three author keywords occur more than 40 times and were automatically classified into five clusters. Following selection, 72 keywords were further analyzed. Cluster 2 had the largest number of keywords (26), followed by cluster 4 (23), and cluster 1 (11). Cluster 1 was the topic of ERAS programs in orthopedic surgery, with the top three most frequent keywords being “rehabilitation”, “total knee arthroplasty”, and “hip fracture”. Cluster 2 was related to the ERAS protocol in gastrointestinal surgery, with the most popular keywords “enhanced recovery after surgery”, “colorectal surgery”, and “complications”. Cluster 3 was associated with clinical nutrition in surgery, with the most frequent keywords being “surgery”, “outcome”, and “nutrition”. Cluster 4 correlated to ERAS programs of pain management, with the most commonly occurring keywords being “pain”, “postoperative”, and “analgesia”. Cluster 5 correlates with ERAS programs of cardiac surgery, with the most used keywords being “fast-track”, “cardiac surgery”, and “recovery” (Table S1, Supplementary Materials).

## 4. Discussion

In the present study, bibliometric analysis was used to find ERAS characteristics from multiple perspectives and further identified the relationship between citations and Mendeley readers in this area.

### 4.1. Principal Findings and Explanation

In this bibliometric analysis, 8539 ERAS-related studies were identified for the period 1994−2019, with the annual global publication showing significant growth since 2014. This condition might be affected by various guidelines published by the ERAS Society in recent years [1,9,10,23,24,25,26,27,28,29,30,31,32,33,34,35,36,37,38,39,40,41,42,43,44]. Eighteen languages were published in ERAS-related studies. With the exception of Korean and Japanese, other languages are typically used in European countries. English is the dominant language for academic communication, accounting for the largest proportion (95.5%).

Ninety-one countries contributed to the ERAS programs. The list of the top 10 largest contributing countries reported that 70% originated in European countries, with others from Northern America (2) and Asia (1). The US is the most influential country with the highest number of publications. Eighty-two countries were involved in international collaborations. The UK had the highest number of international collaborations (52), followed by the US (50), and The Netherlands (44). The list of the top 31 most frequent collaborations between countries shows 13 countries with more than 30 international cooperations. The most frequent collaborative countries were between the US and Canada (132). Approximately 51.6% of international collaborations were between European countries. These phenomena most likely indicate that geographical location is the potential advantage of fostering intercountry collaborations, which were closer between European countries.

The result from the top 10 most productive authors shows anesthesiologists and surgeons to be predominant in ERAS programs [45,46,47,48]. Increasing attention should be paid to the role of nurses and physiotherapists in multidisciplinary work in the future to improve patient outcomes [49,50,51]. Henrik Kehlet is the most relevant and academic influential author in ERAS. Olle Ljungqvist, the chairman of the ERAS society, ranked second. In addition, Henrik Kehlet was also the top active author in this area, contributing research papers commencing annually in 1997 (Figure 3).

Of the top 10 journals with the greatest number of publications, Anesthesia and Analgesia ranked first place. Most ERAS-related studies were published in anesthesiology journals, with more than half of the top 10 journals being anesthesia-related journals. The list of the top 10 highest impact factor journals shows that a high impact and quality medical journal was interested in the ERAS. All journals were in the first quartile, with impact factors above 20. The year of the journal’s first publication in ERAS indicated that ERAS-related research developed rapidly and popularly, with an increasing amount of new journals producing ERAS-related research, with the highest number of new journals being reached in 2018 (Figure 4). ERAS programs were identified in 11 clinical departments from 6920 papers, with 72.7% (eight) of the most relevant journals being in the first quartile. Gastrointestinal, orthopedic, and cardiac surgeries ranked as the top three most popular departments in ERAS.

Citations are a valuable indicator for assessing the quality of literature. The current study discovered the top five articles with the highest academic influence based on the number of citations. As the main disadvantage of citations could not reflect the value of the literature in the early stages, we further analyzed whether the alternative indicator (Mendeley readers) helps readers determine its academic impact. Simple linear regression analysis of 8079 papers presented a significant positive correlation between citations and Mendeley readers, with the latter being able to be potentially put to use as a reference indicator for research quality assessment in ERAS.

The hot topic was determined according to the frequency of the author’s keywords. Five research themes were identified in the present study. Cluster 1 relates to the ERAS programs in orthopedic surgery. Total knee arthroplasty, total hip arthroplasty, and anterior cruciate ligament reconstruction were the most often performed orthopedic surgeries in ERAS programs. Hip fracture was the most frequently occuring orthopedic disease in ERAS pathways. Functional exercise is one of the most critical components in the perioperative management of orthopedics, improving patient outcomes. Based on the guidelines of the ERAS society perioperative care on total hip replacement and total knee replacement surgery, the evidence level and recommendation grade of early mobilization were strong [32]. Cluster 2 is associated with the ERAS programs in gastrointestinal surgery. Surgeries most related to the ERAS programs were bariatric surgery, gastrectomy, colectomy, and pancreaticoduodenectomy, with the laparoscopic instrument being the most frequently applied. Several studies supported laparoscopic use to achieve a better prognosis than open surgery, with laparoscopic use in gastrointestinal surgery appearing to be more consistent with the concept of ERAS [24,52,53,54]. The National Surgical Quality Improvement Program data are commonly used to assess the outcome of ERAS programs [55,56]. Gastrointestinal surgeons, who utilized ERAS programs for their patients, are more focused on readmission, morbidity, mortality, length of stay, and surgery-related intestinal obstruction. Cluster 3 was associated with clinical nutrition in surgery. Malnutrition was associated with increased postoperative complications and mortality, and effective interventions reduce the risk of complications [57,58,59]. Perioperative nutritional support is one of the most important aspects of ERAS programs. As patients with cancer were more likely to have perioperative malnutrition, ERAS programs placed increasing focus on the nutritional status in cancer patients [37,41,60,61,62]. Early oral nutrition is advocated by ERAS programs in postsurgical recovery, with some studies supporting an early oral diet to safe, feasible, and shorter hospital stays than noninterventional groups. However, whether oral nutrition can reduce postoperative complications requires further investigation [63,64,65]. Cluster 4 correlates to perioperative pain management. Multimodal analgesia is the core of pain control in ERAS programs. A combination of various analgesics and anesthesia techniques will help reduce opioid-related side effects [66]. Currently, epidural analgesia is the most frequently researched anesthesia technique in multimodal analgesia, with the most concerning adverse effects being analgesics postoperative nausea and vomiting. Cluster 5 correlates with ERAS programs in cardiac surgery. The first cardiac surgery report dates back to 1994 and was termed “fast-track recovery” to treat coronary artery bypass grafting patients [67]. The development of ERAS-related cardiac surgery matured over the years, and clinical guidelines in cardiac surgery were published by the ERAS society [31].

### 4.2. Implications for Research and Practices

In the present study, bibliometric analysis was used to characterize ERAS research from multiple perspectives. These findings will likely help scholars in their further investigations. First, country information could help provide the researcher with information on the current global contribution of countries, as well as providing a reference for further improve and enhance international cooperation. Second, institution lists provide value information to practitioners who want to accept advanced research training in experienced organizations. Third, author information may improve scholarly communication, as it provides a reference to participation in the ERAS meeting or manuscript review. Fourth, journal information likely aids scholars to further subscribe, trace the most related journal in the future, or submit an ERAS-related manuscript as a reference. Fifth, citations and Mendeley readers could assist scholars in quickly finding high-impact articles in the area of ERAS. Finally, the type of surgery and research topic help physicians discover the mainstream discipline, research hotspot, and inadequate research areas. Hence, it may contribute to improving variations across disciplines and provide reference direction for further studies.

### 4.3. Limitations

The present study has several limitations. First, only a single database was used in this study. Web of Science is the most frequently used database in bibliometric studies, and most of the bibliometric softwares could identify the format from Web of Science [68,69,70,71]. However, the drawback is that some valuable literature from other database sources are most likely missed [13]. Second, the research topic of ERAS contains multiple medical disciplines. Although we selected multiple search terms to identify more relevant research, some potential papers might still be missed. Third, conference proceedings were included in the bibliometric analysis. Therefore, identical content may exist in the literature, as results may be published as a conference abstract as well as a complete journal article [72].

## 5. Conclusions

In summary, our bibliometric study indicates that the overall global contribution shows an increase in ERAS-related research. The US is the most influential country, whereas the UK is the most cooperative country. Henrik Kehlet is the most relevant, academically influential and active author in this field. Anesthesiologists and surgeons were predominant in the ERAS program. The journal Anesthesia and Analgesia had the most related articles, with anesthesiology-related and high-impact medical journals being the most interested in ERAS. Mendeley readers could be used in ERAS research to assess literature quality. ERAS programs were more likely to be utilized for gastrointestinal, orthopedic, and cardiac surgeries. Pain management and perioperative nutrition were more concerned with ERAS programs.

## Figures and Tables

**Table 1:** Language distribution of publications.

Languages	Records
English	8156
German	233
French	71
Spanish	38
Portuguese	11
Korean	6
Turkish	6
Czech	3
Romanian	3
Hungarian	2
Lithuanian	2
Serbian	2
Icelandic	1
Italian	1
Japanese	1
Polish	1
Russian	1
Slovene	1

**Table 2:** Top 10 largest contributing countries in ERAS.

Countries	Records	h-Index	Sum of Times Cited	Average Citations per Item
US	2717	146	107,682	39.59
UK	818	101	41,177	50.22
China	663	51	10,431	16.15
Germany	626	76	25,574	40.79
Canada	599	87	29,740	49.57
Denmark	477	89	31,092	65.05
Italy	432	65	20,225	46.71
The Netherlands	360	71	21,898	60.66
Sweden	357	76	21,619	60.39
France	335	57	12,792	38.19

**Table 3:** Top 31 most frequent collaborating countries.

From	To	Frequency
US	Canada	132
US	UK	98
US	Germany	71
UK	Canada	66
UK	Sweden	62
UK	Germany	58
US	China	57
US	Italy	57
The Netherlands	UK	55
US	Switzerland	53
UK	Italy	50
Italy	Germany	49
UK	Switzerland	47
The Netherlands	Germany	42
Sweden	Canada	42
Switzerland	Germany	41
US	Sweden	41
UK	Australia	39
US	The Netherlands	39
US	Denmark	37
Switzerland	France	36
UK	Norway	36
UK	France	35
US	France	34
Sweden	Switzerland	33
The Netherlands	Italy	32
US	Australia	32
Canada	Germany	31
France	Germany	31
Italy	Switzerland	31
UK	Denmark	31

**Table 4:** Top 10 authors with the greatest number of publications in ERAS.

Authors	Articles	Sum of Times Cited	h-Index	Specialized Subject	Institution	Country
Kehlet, H.	261	21,497	74	Surgical Pathophysiology	Copenhagen Univ Hosp	Denmark
Ljungqvist, O.	99	11,877	52	Department of Surgery	Orebro University	Sweden
Carli, F.	88	5607	41	Department of Anesthesiology	McGill University	Canada
Demartines, N.	81	4413	27	Department of Visceral Surgery	Centre Hospitalier Universitaire Vaudois	Switzerland
Hubner, M.	63	2346	23	Department of Visceral Surgery	Centre Hospitalier Universitaire Vaudois	Switzerland
Gan, T.J.	57	4494	33	Department of Anesthesiology	Stony Brook University	US
Husted, H.	57	2717	29	Department of Orthopedic Surgery	Copenhagen Univ Hosp	Denmark
White, P.F.	55	2952	32	Department of Anesthesiology	Cedars Sinai Medical Center	US
Feldman, L.S.	52	2045	23	Department of Surgery	McGill University	Canada
Lobo, D.N.	48	6078	30	Department of Gastrointestinal Surgery	University of Nottingham	UK

**Table 5:** Top 10 journals ranked by the number of publications and impact factor, respectively.

Source	Journal Impact Factor	Journal Quartile	Number of Publications	Total Citations	h-Index
Anesthesia and Analgesia	4.305	Q1	358	21,494	79
British Journal of Anaesthesia	6.880	Q1	189	14,386	65
Acta Anaesthesiologica Scandinavica	2.050	Q3	147	5060	40
Surgical Endoscopy	3.149	Q1	147	4213	37
Anesthesiology	7.067	Q1	135	12,697	65
World Journal of Surgery	2.234	Q2	128	4817	36
European Journal of Anaesthesiology	4.500	Q1	127	3564	29
Journal of Cardiothoracic and Vascular Anesthesia	2.258	Q3	125	3101	31
Annals of Surgery	10.130	Q1	117	11,928	59
Colorectal Disease	2.769	Q2	111	3328	32
The New England Journal of Medicine	74.699	Q1	2	839	2
Lancet	60.392	Q1	10	5118	10
The Journal of the American Medical Association	45.540	Q1	8	2637	8
Lancet Oncology	33.752	Q1	2	267	2
Journal of Clinical Oncology	32.956	Q1	3	289	3
British Medical Journal	30.223	Q1	8	1451	8
Nature Reviews Gastroenterology and Hepatology	29.848	Q1	2	66	2
Lancet Infectious Diseases	24.446	Q1	1	24	1
Circulation	23.603	Q1	7	764	7
European Heart Journal	22.673	Q1	3	222	3

**Table 6:** Type of surgery and most relevant journals.

Subject	Articles	Journal Title	Articles	Impact Factor	Journal Quartile
Gastrointestinal surgery	2162	Surgical Endoscopy	110	3.149	Q1
Orthopedic surgery	1740	Journal of Arthroplasty	85	3.709	Q1
Cardiac surgery	608	Journal of Cardiothoracic and Vascular Anesthesia	99	2.258	Q3
Thoracic surgery	462	Journal of Thoracic Disease	55	2.046	Q3
Head and neck surgery	438	Anesthesia and Analgesia	22	4.305	Q1
Obstetrics and gynecology surgery	419	Anesthesia and Analgesia	40	4.305	Q1
Urologic surgery	385	Urology	35	1.924	Q3
Hepatobiliary surgery	359	Surgical Endoscopy	25	3.149	Q1
Pancreatic surgery	180	HPB	18	3.401	Q1
Breast surgery	143	Plastic and Reconstructive Surgery	22	4.209	Q1
Vascular surgery	24	Journal of Vascular Surgery	3	3.405	Q1

**Table 7:** Top 5 highest number of citations publication in ERAS.

First Author	Article Title	Source Title	Times Cited	Publication Year
Kehlet, H.	Persistent postsurgical pain: risk factors and prevention	Lancet	1923	2006
Kehlet, H.	Multimodal approach to control postoperative pathophysiology and rehabilitation	British Journal of Anaesthesia	1121	1997
Heran, B.S.	Exercise-based cardiac rehabilitation for coronary heart disease	Cochrane Database of Systematic Reviews	1105	2011
Falck-Ytter, Y.	Prevention of VTE in Orthopedic Surgery Patients Antithrombotic Therapy and Prevention of Thrombosis, 9th ed: American College of Chest Physicians Evidence-Based Clinical Practice Guidelines	Chest	970	2012
Brandstrup, B.	Effects of intravenous fluid restriction on postoperative complications: Comparison of two perioperative fluid regimens—A randomized assessor-blinded multicenter trial	Annals of Surgery	970	2003

## Data Availability

Not applicable.

## Supplementary Materials

The following are available online at https://www.mdpi.com/1010-660X/57/2/172/s1, Table S1: Research topic of ERAS.
