# Supplementary material for: The Pertinent Literature of Enhanced Recovery after Surgery Programs: A Bibliometric Approach
Source: Medicina (Kaunas). 2021 Feb 17;57(2):172. doi: 10.3390/medicina57020172 (PMC7922786; doi:10.3390/medicina57020172)
Supplement: Supplementary file 1 [file medicina-57-00172-s001.pdf]

Cluster 1	
**Occurrences**	**Words**
250	rehabilitation
120	total knee arthroplasty
66	hip fracture
63	elderly
61	total hip arthroplasty
51	enhanced recovery after surgery (eras)
51	quality of life
49	patient
43	randomized controlled trial
43	anterior cruciate ligament
42	exercise

Cluster 2	
**Occurrences**	**Words**
379	enhanced recovery after surgery
297	colorectal surgery
261	complications
200	length of stay
199	laparoscopy
156	perioperative care
154	outcomes
135	fast-track surgery
122	postoperative complications
107	colorectal cancer
106	meta-analysis
90	colorectal
75	NSQIP
75	morbidity
74	bariatric surgery
67	mortality
64	gastric cancer
64	postoperative ileus
56	gastrectomy
53	fast track surgery
52	readmission
51	quality improvement
45	postoperative recovery
45	rectal cancer
44	colectomy
42	pancreaticoduodenectomy

Cluster 3	
**Occurrences**	**Words**
476	surgery
97	outcome
78	nutrition
65	cancer
64	perioperative
56	prehabilitation
46	nursing
43	oral nutrition

Cluster 4	
**Occurrences**	**Words**
261	pain
246	postoperative
229	analgesia
220	postoperative pain
146	anesthesia
77	propofol
76	pain management
71	epidural analgesia
70	remifentanil
68	opioids
65	postoperative analgesia
63	postoperative nausea and vomiting
58	dexmedetomidine
58	dexamethasone
57	ropivacaine
54	vomiting
53	bupivacaine
53	sevoflurane
52	morphine
50	ketamine
46	multimodal analgesia
44	fentanyl
43	nausea

Cluster 5	
**Occurrences**	**Words**
161	fast-track
112	cardiac surgery
109	recovery
79	postoperative care
